# Supplementary material for: A Quantitative Analysis Model Established to Determine the Concentration of Each Source in Mixed Astaxanthin from Different Sources
Source: Molecules. 2020 Jan 31;25(3):628. doi: 10.3390/molecules25030628 (PMC7036820; doi:10.3390/molecules25030628)
Supplement: Supplementary file 1 [file molecules-25-00628-s001.pdf]

Figure Captions

Figure No.	Caption
Figure S1	The calibration curves of (3R, 3'R)-astaxanthin (A) and (3S, 3'R)-astaxanthin (B) from *P. rhodozyma*.
Figure S2	The calibration curves of (3S, 3'S)-astaxanthin (A) and (3S, 3'R)-astaxanthin (B) from *H. pluvialis*.
Figure S3	The calibration curves of (3S, 3'R)-astaxanthin (A), (3R, 3'R)-astaxanthin (B) and (3S, 3'S)-astaxanthin (C) from synthetic astaxanthin.

Figure S1

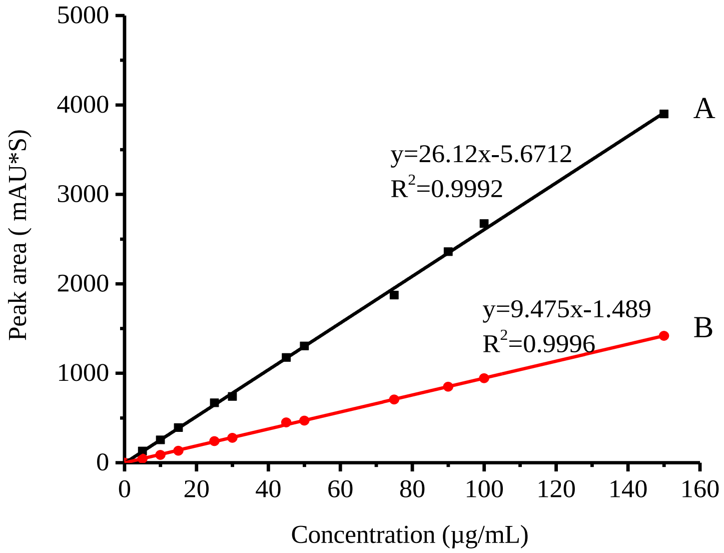

Peak area (mAU*S)
5000
4000
3000
2000
1000
0
0
20
40
60
80
100
120
140
160
Concentration (μg/mL)
y=26.12x-5.6712
R2=0.9992
A
y=9.475x-1.489
R2=0.9996
B

Figure S2

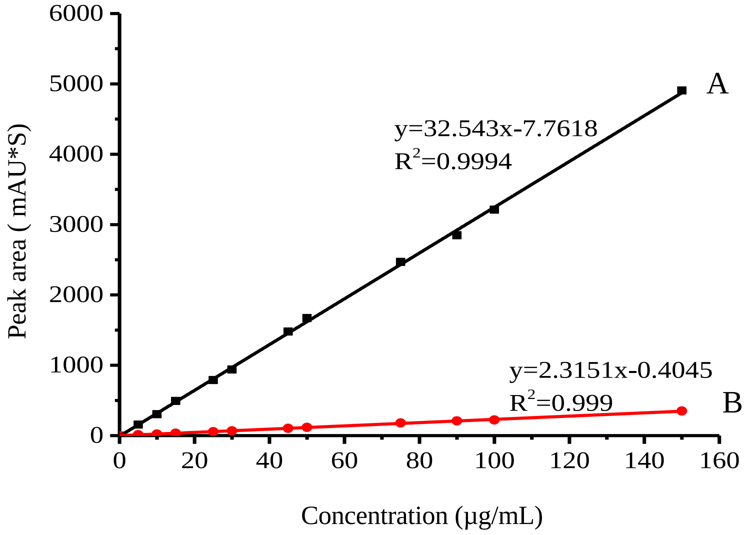

6000
5000
4000
3000
2000
1000
0
Peak area (mAU*S)
A
y=32.543x-7.7618
R2=0.9994
y=2.3151x-0.4045
R2=0.999
B
0
20
40
60
80
100
120
140
160
Concentration (μg/mL)

Figure S3

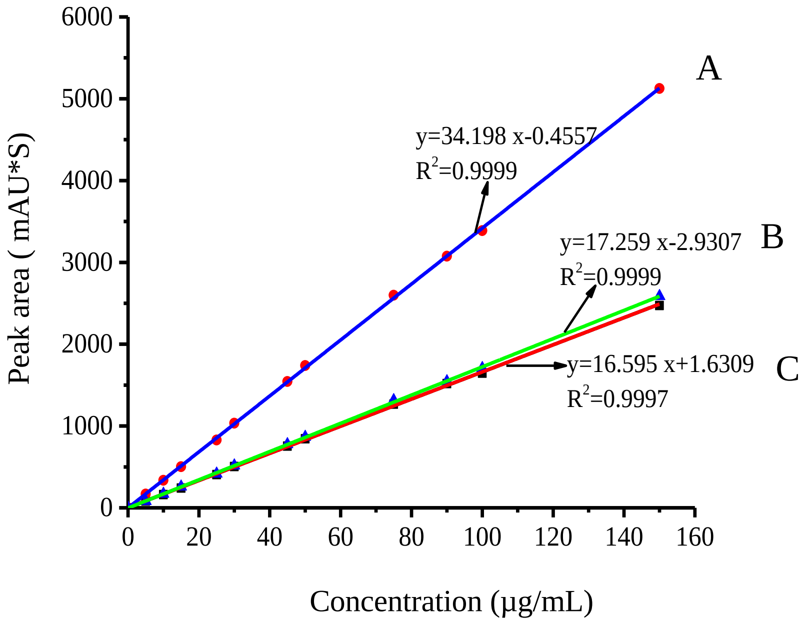

6000
5000
4000
3000
2000
1000
0
Peak area (mAU*S)
A
y=34.198 x-0.4557
R2=0.9999
B
y=17.259 x-2.9307
R2=0.9999
C
y=16.595 x+1.6309
R2=0.9997
0
20
40
60
80
100
120
140
160
Concentration (μg/mL)
